# Cost-effectiveness of district-wide seasonal malaria chemoprevention when implemented through routine malaria control programme in Kita, Mali using fixed point distribution

**DOI:** 10.1186/s12936-021-03653-x

**Published:** 2021-03-04

**Authors:** Halimatou Diawara, Patrick Walker, Matt Cairns, Laura C. Steinhardt, Fatou Diawara, Beh Kamate, Laeticia Duval, Elisa Sicuri, Issaka Sagara, Aboubacar Sadou, Jules Mihigo, Erin Eckert, Alassane Dicko, Lesong Conteh

**Affiliations:** 1 Malaria Research & Training Centre, Faculty of Pharmacy and Faculty of Medicine and Dentistry, University of Sciences Techniques and Technologies of Bamako, P.O Box 1805, Bamako, Mali; 2 Department of Infectious Disease Epidemiology, School of Public Health, Imperial College London, London, UK; 3 London School of Hygiene & Tropical Medicine, London, UK; 4 Malaria Branch, Division of Parasitic Diseases and Malaria, Center for Global Health, Centers for Disease Control and Prevention, 1600 Clifton Road NE, Mailstop H24-3, Atlanta, GA 30329 USA; 5 Maternal and Child Survival Program, Save the Children, Bamako, Mali; 6 President’s Malaria Initiative, US Agency for International Development, Kinshasa, Democratic Republic of the Congo; 7 President’s Malaria Initiative, US Agency for International Development, Bamako, Mali; 8 President’s Malaria Initiative, USAID Bureau for Global Health, Office of Health, Infectious Diseases, and Nutrition, 2100 Crystal Drive, Arlington, VA 22202 USA; 9 Department of Health Policy, London School of Economics and Political Science, Houghton Street, London, WC2A 2AE UK

**Keywords:** Malaria, Mali, Seasonal malaria chemoprevention, Cost, Cost-effectiveness, Economic, Financial, Disability-adjusted life year (DALY)

## Abstract

**Background:**

Seasonal malaria chemoprevention (SMC) is a strategy for malaria control recommended by the World Health Organization (WHO) since 2012 for Sahelian countries. The Mali National Malaria Control Programme adopted a plan for pilot implementation and nationwide scale-up by 2016. Given that SMC is a relatively new approach, there is an urgent need to assess the costs and cost effectiveness of SMC when implemented through the routine health system to inform decisions on resource allocation.

**Methods:**

Cost data were collected from pilot implementation of SMC in Kita district, which targeted 77,497 children aged 3–59 months. Starting in August 2014, SMC was delivered by fixed point distribution in villages with the first dose observed each month. Treatment consisted of sulfadoxine-pyrimethamine and amodiaquine once a month for four consecutive months, or rounds. Economic and financial costs were collected from the provider perspective using an ingredients approach. Effectiveness estimates were based upon a published mathematical transmission model calibrated to local epidemiology, rainfall patterns and scale-up of interventions. Incremental cost effectiveness ratios were calculated for the cost per malaria episode averted, cost per disability adjusted life years (DALYs) averted, and cost per death averted.

**Results:**

The total economic cost of the intervention in the district of Kita was US $357,494. Drug costs and personnel costs accounted for 34% and 31%, respectively. Incentives (payment other than salary for efforts beyond routine activities) accounted for 25% of total implementation costs. Average financial and economic unit costs per child per round were US $0.73 and US $0.86, respectively; total annual financial and economic costs per child receiving SMC were US $2.92 and US $3.43, respectively. Accounting for coverage, the economic cost per child fully adherent (receiving all four rounds) was US $6.38 and US $4.69, if weighted highly adherent, (receiving 3 or 4 rounds of SMC). When costs were combined with modelled effects, the economic cost per malaria episode averted in children was US $4.26 (uncertainty bound 2.83–7.17), US $144 (135–153) per DALY averted and US $ 14,503 (13,604–15,402) per death averted.

**Conclusions:**

When implemented at fixed point distribution through the routine health system in Mali, SMC was highly cost-effective. As in previous SMC implementation studies, financial incentives were a large cost component.

## Background

After an unprecedented period of success, with substantial investment in malaria control and reductions in malaria episodes, the progress has stalled [1]. In 2018, there were an estimated 228 million cases and 405,000 related deaths worldwide, with a high proportion of these deaths occurring in sub-Saharan Africa (93%) mostly in children under 5 years [1].

In addition to the human morbidity and mortality impact, malaria places economic burdens on individuals, health systems and national governments. In Mali, malaria is the main cause of outpatient visits, hospitalizations and mortality in health facilities; children under five and pregnant women are the most affected [2]. According to a national survey in 2013, the prevalence of malaria parasitaemia by microscopy was 52% in children under 5 years overall in Mali and 37% in the southern region of Kayes, the setting of this study [3]. In 2014, health facilities across Mali registered more than two and half million cases of suspected malaria, of which 1.7 million were clinical cases, 800,000 severe cases and 2309 deaths [4].

Seasonal malaria chemoprevention (SMC) was recommended by the World Health Organization (WHO) in areas of highly seasonal malaria transmission across the Sahel sub-region in 2012. It consists of the administration of a complete treatment course of sulfadoxine-pyrimethamine and amodiaquine (SP + AQ) at monthly intervals to children aged 3 to 59 months, beginning before the start of the transmission season, with up to a maximum of four doses during transmission season [5]. The National Malaria Control Programme (NMCP) in Mali was one of the early adopters and introduced SMC in 2012 with pilot implementation in one district and progressive expansion to other districts.

Few studies have assessed the cost and cost-effectiveness of SMC introduction and distribution at scale through the routine health system. In addition, little is known about the detailed cost breakdown of SMC when implemented using fixed point distribution method [6, 7]. Peer-reviewed cost and cost-effectiveness studies using data collected alongside clinical trials and modelling efforts have suggested that SMC is a low cost and highly effective intervention (Table 1). The lowest cost per child per dose reported was in Senegal at $1.96 for 3 doses (inflated to 2016 USD) [7] and the highest cost per dose at $ 22.81 in the upper west region in Ghana (inflated to USD 2016) [6]. Cost drivers included the cost of the drugs, scale of the study, the delivery mode (fixed point vs*.* door-to-door), personnel costs (salaries) and incentives. Incentives reflect a payment (either in kind or in monetary terms) to an individual independent of any salary they may receive, for efforts carried out in addition to their routine activities.

**Table 1:** Summary of peer reviewed SMC cost and cost-effectiveness estimates

Setting	Delivery strategy	Unit Cost/US $ (2016)^a^						Cost–effectiveness ratios^b^
		Cost per round			Cost per fully adherent child^c^			
		Financial		Economic	Financial		Economic	
Hohoe, Ghana (Conteh et al. 2010)	DTD, 6 rounds, AS + AQ monthly – trial conditions DTD, 6 rounds, AS + AQ monthly – modelled to district level	1.84		2.54	13.17 4.02		16.70 4.83	80.46 (66.18–94.81) per case averted 24.87 (22.63–27.36) per case averted
Basse, Gambia (Bojang et al. 2011)	FPD, 3 rounds, SP + AQ monthly DTD, 3 rounds, SP + AQ monthly	1.04 0.74		1.20 0.97	3.31 1.37		3.87 1.82	Not applicable
Jasikan, Ghana (Patoulllard et al. 2012)	FPD, 3 rounds SP + AQ monthly DTD, 3 rounds, SP + AQ monthly	2.93–3.09 2.65		3.65–3.79 3.46	– –		9.37 8.32	Not undertaken
Upper West Region, Ghana (Nonvignon et al. 2016)	DTD, 4 rounds, SP + AQ at monthly						22.81	108.41 (101.01–123.01) per additional case averted^d^ 3339.97 (3112.03–3789.85) per additional death averted
Bambey, Mbour, Fatick & Niakhar, Senegal (Pitt et al., 2017)	DTD, 3 rounds SP + AQ monthly	0.55		0.65	1.65		1.96	Not undertaken
Burkina Faso, Chad, The Gambia, Guinea, Mali, Niger, and Nigeria (ACCESS-SMC Partnership, 2020)	DTD and FPD (country dependent) 4 rounds SP + AQ monthly	–		0.91^e^	–		–	2.91–30.73 per case averted 119.63- 506.00 per severe case averted 533.56- 2256.92 per death averted

DTD: Door to Door distribution, FPD: Fixed Point Distribution (Health facility, outreach or focal point in village)

^a^Costs from Provider perspective and inflated from original source base year to 2016 using Inflation Calculator from U.S. Labor Department's Bureau of Labor Statistics on September 12, 2019

^b^Based on intervention costs only

^c^The unit cost per round weighted by adherence levels

^d^Nonvignon et al. (2016) used a similar approach to this study (combined primary cost data with a transmission model to estimate cost-effectiveness)

^e^According to the manuscript, ‘The weighted average cost of four treatments per child was obtained by dividing the total recurrent cost by the total number of doses administered divided by 4′ (p1834). This amounted to US $3.63, a calculation based on a multiple of treatment rounds that appears to exclude adherence. To obtain the cost per round, US $3.63 was divided by 4. Mali specific costs were not presented

There have been two implementation studies of SMC in Mali [8, 9]. In 2012, an evaluation of SMC was undertaken by “Médecins Sans Frontières” (MSF) in Koutiala, the first Malian health district to implement SMC. Using budgetary estimates, the average cost of providing SMC to a child was estimated at $1.30 per child per round [8]. In addition, Mali was part of a large multi-county rapid expansion of seasonal malaria chemoprevention in the Sahel (ACCESS SMC) implementation study [10]. Preliminary ACCESS SMC cost estimates suggested that the recurrent costs of delivering 4 doses of SMC to a child in Mali was $4.05 (2015 USD) [11]. In a later publication, an average economic cost for four treatments per child, across all seven ACCESS countries, of US $ 3.63 (US $ 2.71 to US $ 8.20) was obtained [12]. To date, this is the first study to use primary cost data to estimate the cost per disability-adjusted life year (DALY) averted for SMC.

SMC is a relatively new approach compared to other malaria control tools. Therefore, rigorous evaluations of the strategy when implemented through a routine health system, outside the context of clinical trials, are needed to inform resource allocation decisions. Often trials are well funded and able to incentivize those involved in ensuring the delivery of SMC, both in terms of financial payments and non-financial incentives such as additional training and improved access to drugs and other resources. The aim of this evaluation was three-fold: firstly, to assess the district-wide primary cost data associated with the implementation of SMC in Kita as part of routine care; secondly to combine these costs with coverage levels identified in the project to estimate the effectiveness of SMC in Kita based upon an established dynamic malaria transmission model. Thirdly, to use the costs and the effectiveness estimates to calculate the incremental cost-effectiveness of the intervention compared to no intervention.

## Methods

### Study site and population

Kita district, where SMC was implemented and evaluated in 2014, is located in the western region of Kayes, about 180 kms north of Bamako, the capital city of Mali. Kita was one of the 21 districts in Mali to roll out SMC in 2014 as part of the progressive national scale up of SMC. Kita has one district hospital, 40 community health centres, and 72 community health workers (CHWs). The population was estimated to be 516,649 in 2014 with approximately 77,497 children between 3 and 59 months of age and parasitaemia prevalence for the region in 2013 was 37% [13, 14]. SMC was implemented in all 336 villages/quartiers and while approximately 77,497 children were forecast to receive SMC, an estimated 104,255 children actually received the intervention. This higher number of children than expected is explained by three factors. Firstly, some children older than 5 years are likely to have received the intervention; secondly, Bafoulabe, the district next to Kita did not receive SMC and it is likely that children came from bordering villages to receive SMC; and finally, the study site covered a large agricultural area that, at the time of SMC administration, might have experienced a population swell given it was growing season so there was a need for increased labour.

### SMC delivery and administration

SMC delivery refers to the process of obtaining the resources (mainly drugs and incentives) from the central stores to the end user (in this case the child). In the context of this study, incentives capture the financial compensation for supporting for SMC implementation in Kita. SMC administration (sometimes referred to in previous SMC costing papers as distribution) refers to the activities associated with giving the drugs to the child on the day(s) of SMC.

In Kita, the SMC campaign was organized in 2014 using a fixed point distribution method. Specifically, drug distribution was channelled through the district hospital and community health centres, and SMC was delivered at village focal points. Distribution was the responsibility of government employed health staff, primarily nurses and community health workers (CHWs).

Children aged 3–59 months received sulfadoxine-pyrimethamine and amodiaquine (SP + AQ) at monthly intervals over the four months of August, September, October, and November 2014. Before drug distribution started, several meetings were held from May to August 2014 at the national, regional, district and sub-districts levels in Kita in preparation for SMC implementation. Two technical groups composed of 8 staff members from NMCP and implementing partners were created: one to establish, review and update the SMC distribution modules, and develop data collection tools; and the second group to develop a communication plan for SMC implementation. SMC dispensers, who were CHWs and nurses, received a two-day training course on SMC administration using the training modules developed. Normally, the Central Pharmacy (Pharmacie Populaire du Mali, PPM) provides drugs and medical supplies to the Regional Health Directorate in Kayes. The Regional Directorate then provides supplies and drugs to health districts who dispatch them to the district and Community Health Centres. However, because of a delay in preparing for implementation, on this occasion SMC drugs were paid for by the Ministry of Health (MoH) through the NMCP and delivered directly to the district heath centre of Kita by the PPM without passing through the Regional Health Directorate. It then took five days for the district health centre to deliver the drugs and supplies to community health centres.

The SP + AQ used for SMC in Kita were not co-blistered or pre-packed conditional on age group. The drugs, therefore, had to be cut and repacked by the district health team. For this, 10 persons were recruited and worked on the drugs packaging for 25 days. After the drugs were packaged, an allocation plan was developed to distribute the SMC drugs and necessary supplies for implementation to the community health centres and villages heath workers. Information, sensitization and communication messages on SMC were developed and delivered through the two most popular local radio channels for six months (July to December 2014). Additional specific messages were developed and added during the distribution period. In addition, at the community level, mobilization and sensitization activities were carried out.

SMC drug administration was performed by Kita district health staff and comprised 588 drug dispensers (nurses and CHWs) organized into 133 teams of 2–6 health workers each at health centres (n = 37 teams) and village fixed points (n = 96 teams). The first dose of AQ and the single dose of SP was given the first day by health workers. Children were observed for 30 min, and if the child vomited within this time, another dose was administered. The second and third doses of AQ were given to parents to be administrated at home. Further details are provided elsewhere [3].

All labour used in SMC distribution was employed and paid by the MoH, except implementing partner staff who were paid by the non-government organization Save the Children [12]. Supervision and monitoring were performed during the distribution period by teams composed of staff at national level ((NMCP, Maternal and Child Survival Programme, and National Federation for Community Health Centres Association)), regional level and district level. As with all SMC programmes, incentives were given to all of those involved: NMCP personnel, Fédération Nationale des Associations Communautaires, staff from the district hospital and community health centres, CHWs and drivers. Incentives (per-diems and accommodation where required) varied according to categories of the staff and location (local, district level, regional). In Kita incentives were set at daily rates comparable to financial incentives paid for delivering other interventions and research studies conducted in the area previously. The daily incentive for a rural doctor was comparable to their daily salary, and a little less than a CHW could expect to receive for their daily duties. On average those involved in the implementation were paid approximately 36 days of incentives for helping with SMC implementation in 2014.

### Costing

Costs are presented from the provider perspective using an ingredients approach whereby the relevant resources were identified and measured at the time of the SMC implementation to estimate the total cost of the intervention [5]. The choice of provider perspective was based on data availability and the intention of the study to inform decision makers how much the intervention would cost for funding proposes. Both financial and economic costs (recurrent and capital) are estimated for the 4 rounds of distribution. Financial costs reflect the actual expenditure required to deliver the intervention such as the cost of drugs and incentives. Economic costs capture the opportunity cost of all resources used to provide SMC, whether or not they incur a financial cost. For example, the time of health personnel involved in SMC delivery represents an economic cost as the staff already received a salary so there was no additional financial commitment, however they could have spent their time on other activities, so we need to capture the opportunity cost [6, 15]. Only 2014 cost data were used in this study and are presented in 2016 Communauté Financière en Afrique (CFA) and US dollars (USD). A conversion rate of CFA 494.17: USD 1 was assumed based on the average of August 2014 inflated to 2016 using US Inflation Calculator [16].

Table 2 presents the cost categories used in the analysis: planning, communication, training, drugs, personnel, equipment and transport. In addition to the total implementation cost and the total cost per round, unit costs are estimated in terms of cost per child fully adherent (i.e. a child that had the first of each of the SMC drugs under observation in all 4 rounds) and cost per child partially covered (i.e. a child that had the first SMC drugs under observation in 1, 2 or 3 rounds). *Fully* adherent is defined as child who received all 4 rounds and *highly* adherent as child who received at least 3 rounds.

**Table 2:** Cost categories

Cost category Input or Activity	Description	Sample size	Data source
Planning meetings:	In total six meetings were held, including five field visits	6 meetings	MCSP expenses record
Training	A group of trainers was trained on SMC distribution. In turn, community health center staff, community and voluntary health workers (n = 100) were trained by trainers (n = 9). Total training cost was estimated using the number of participants and daily per diems; accommodation, refreshments and transportation costs were also included	6 trainings	MCSP expenses record
Drugs	Quantities were estimated by age group from NMCP 2014 drug accountability plan Number of drugs received is from the drug accountability plan	AQ: 179,627 packs of 6 tablets SP: 359 boxes of 1000 tablets	Costs were collected from Pharmacie Populaire du Mali drug stock inventory state, ACT, RDT, and anti- malarial store in November 2014
Personnel	Personnel costs were collected and calculated in planning, training and distribution categories and captured both those directly responsible for delivering SMC and those supervising. Staff costs were classified by specialty; salary (plus associated benefits such as pensions, housing allowance etc.) Per diems and incentives were calculated according to activity	588	Ministry of Health personnel pay slips for 2014
Equipment	The costs of materials and equipment such as drinking glasses, buckets, chairs and tables used during dispensing SMC		MCSP expenses record
Transport	For transport cost during meetings, trainings and drugs distribution, MCSP/SC vehicles were used as well as rented vehicles. Vehicles maintenance costs were included in renting costs		The total number of vehicles used and the costs were estimated from vehicle renting cost per day from the Ministry of Health vehicle renting documents Fuel costs were extracted from MCSP/SC monthly activities accounts

*NMCP* National Malaria Control Program, *MCSP/SC* Maternal and Child Survival Program

### Effectiveness

An aim of this district-wide SMC delivery was to see how feasible the intervention was at scale outside of trial conditions. The project focused on coverage and collected limited data on health outcomes (specifically prevalence of malaria and anaemia) [13]. Instead, modelling was used to provide an indication of the likely effectiveness of the intervention in Kita. An established dynamical transmission model was used [17]; this model has previously been used for a range of applications including to establish global investment targets for the WHO Global Technical Strategy [18]. Full details, as well as source code and instructions for compilation and running, of the model are available elsewhere [19, 20]. In brief, the model is individual-based and non-spatial, with rainfall driving seasonal patterns of mosquito emergence and subsequent patterns of the entomological inoculation rate, incidence of malaria and prevalence of infection, which then determine the level of onwards transmissibility to the mosquito. The human component of the model incorporates heterogeneity in exposure and both acquired and age-dependent immunity, which affects (i) the probability of developing severe and clinical disease, (ii) the duration of detectable blood-stage infection and (iii) onwards infectivity to the mosquito. This allows the model to capture the relationship between transmission and age-dependent patterns of prevalence and disease across a wide range of settings and to simulate the onwards impact of multiple interventions upon transmission (though the magnitude of this impact within the model is limited when SMC is only provided to under 5 s [21]. The model has also been shown to replicate seasonal patterns of malaria burden, driven by rainfall [22] and the effectiveness of SMC within trials (Cairns et al., pers. commun.), to a high degree of accuracy. Here the model was calibrated to patterns of seasonality, based upon rainfall gauge data between 2002 and 2008 in the region, smoothed by Fourier transformation, to provide typical patterns of rainfall throughout the year. Long-lasting insecticidal nets (LLIN) use within the area was also captured by the model, assuming a linear increase from 0% in 2000 to 56.9% observed in Kayes region in 2013 [3] which had risen to 65.2% in 2015 [2] following the universal coverage campaign in 2014. Usage immediately following the 2014 campaign (which was not measured) was calibrated to achieve 65% usage in the area at the end of 2015 (when the above survey was done), with no further assumed distribution in 2015–2017. The model assumes a constant per-capita rate of attrition in usage in the absence of receiving a new net of 0.2 per year [23]. Simulations were then conducted using 1000 parameter sets representing separate draws from the joint posterior distribution of the underlying transmission model. Baseline vectorial capacity in each simulation was calibrated to 2014 dry-season prevalence according to a triangle distribution with mode of 24.1% and a 95% interval between 20.7% and 27.8%, based upon the mean and binomially-distributed 95% confidence intervals (CIs) observed in this survey, and further accounting for temporal trends in seasonality, LLIN usage and insecticidal coverage.

**Fig. 1:**
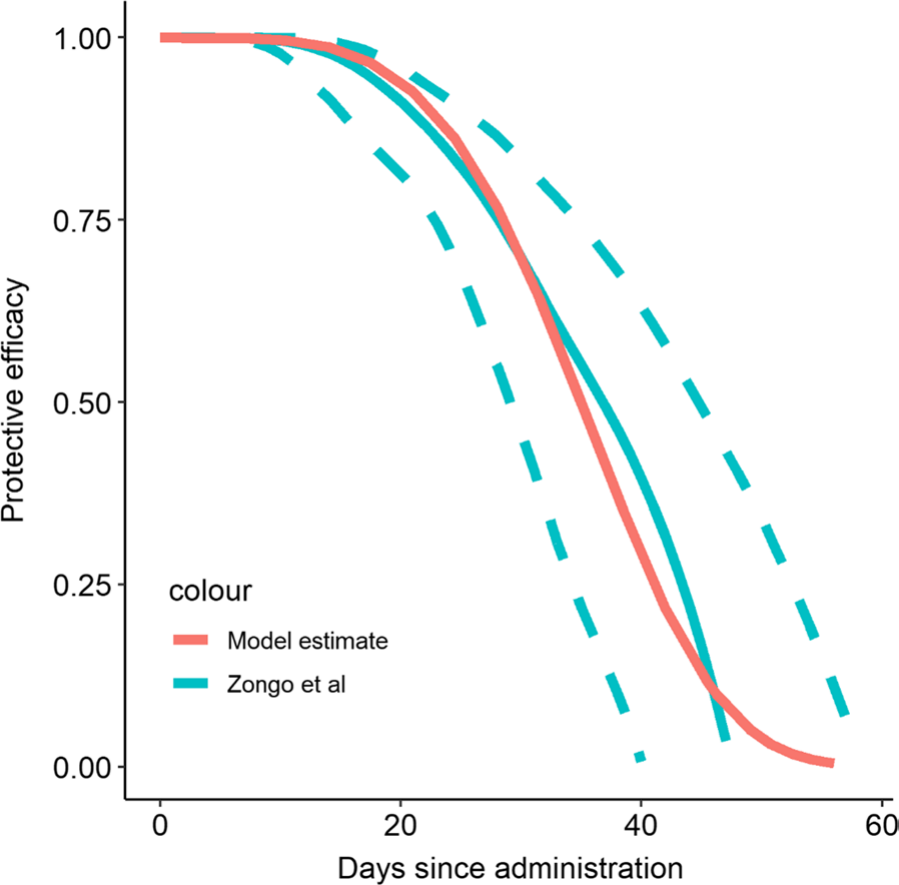
Figure shows the estimated protective efficacy from time since receiving a round of SMC as estimated in [reference 24] compared to the profile used within the model

Four rounds of SMC with SP + AQ were simulated, with a duration of prophylaxis following a Weibull distribution with scale parameter 38.1 and shape parameter 4.3 fitted to published point estimates of the protective efficacy over time from a trial in Burkina Faso [20] (see Fig. 1). This profile provides strong protection for the first 3–4 weeks after a round, declining to 50% protection around 5 weeks, and then subsequently declining rapidly. The model was calibrated to match the per-capita number of doses delivered during the intervention by setting the coverage of the intervention within the model to the overall probability of receiving a dose in any given round according to interviews with the caregivers of children who reported receiving the intervention in 3396 of 4505 (75.3%) potential per-protocol rounds within the survey [13]. Uncertainty in the timing of these doses was then captured by varying the timing of the intervention relative to the seasonal peak by summarising simulations using 1000 draws from a triangle distribution with mode centred around the seasonal peak in transmission, with extremes between a month before and after this optimal scheduling.

Goodness-of-fit was assessed by comparing the model prevalence to prevalence survey collected at the end of 2012 [3] and a month after the final round of SMC [13]. Burden outputs considered were uncomplicated clinical malaria and severe malaria in children under five years and in the wider population, with estimates of lives saved based upon a case-fatality rate of 0.3% [24] per malaria case. The modelling incorporated the universal LLIN campaign within the area in 2014; as a result, in addition to our estimates of 2014 impact, were present both the estimates of the impact of SMC in 2014 specifically, and estimates if SMC at the same coverage as in 2014 had been extended across the 3-year LLIN distribution cycle, in order to provide a measure of the likely effectiveness of SMC if applied continuously in the area.

### Incremental cost effectiveness analysis

An incremental cost effectiveness ratio (ICER) was calculated by dividing the annual total economic cost of SMC by the annual modelled estimates of (1) the number of uncomplicated and severe malaria cases averted, (2) the DALYs averted, and (3) the deaths averted. The annual number of episodes averted was an average of a three-year cycle where the effects of LLINs were also included. DALYs are a measure of burden of disease and can be used as a summary measure to determine and compare the cost-effectiveness of different types of interventions across different diseases and settings [25, 26]. They allow for a more informed assessment of cost effectiveness in relation to thresholds and acceptability. There is no gold-standard for when an intervention is deemed cost-effective. Indeed, the use and mis-use of thresholds is much debated [27, 28]. In helping interpret whether SMC was cost effective we present our findings using two very conservative thresholds: (i) US $780.51, Mali’s 2016 Gross Domestic Product (GDP) per capita and (ii) an even more stringent World Bank threshold of US $250 per DALY averted [29, 30].

### Ethical considerations

The study protocol was reviewed and approved by the Ethical Committee of the Faculty of Medicine, Pharmacy and Dentistry of the University of Bamako prior to the study. The clinical trial was registered at Clinicaltrials.gov as number NCT02894294.

## Results

### SMC cost

The total financial cost to dispense SMC to the 104,255 children in Kita was US $316,111 and the total economic cost was US $357,494. The main economic costs by input were drugs (34%) and personnel (31%). The bulk of personnel costs were due to $81,455 spent on incentives (23% of the total economic costs) (Table 3). The most costly activity was the end stage, dispensing the drugs to the children (Table 4). Financial and economic cost were similar, the main difference being the already covered salary costs of facility-based health staff that were included in economic costs.

**Table 3:** SMC implementation in Kita: Total annual financial and economic costs by input of 2016 USD

	Financial cost		Economic cost	
	$	%	$	%
Personnel	88,429	28	110,729	31
Drugs	122,060	39	122,060	34
Transport	36,190	11	36,190	10
IEC^b^	6584	2	6584	2
Equipment	35,393	11	35,392	10
Other	27,454	9	46,538	13
Total	316,111	100	357,494	100

^a^PPM Central Pharmacy/Pharmacie Populaire du Mali

^b^Information Education & Communication

**Table 4:** SMC implementation in Kita: Total annual financial and economic costs by activity of (2016 USD)

Activity	Financial cost			Economic cost		
	CFA	$	Percentage of total	CFA	$	Percentage of total
Planning meetings	2,305,217	3006	1.39%	2,887,971	5,925	1.66%
*SP-AQ Tablets*		*100,325*			*100,325*	
*Drug Packing*		*5,823*			*5,823*	
*PPM Fees*^a^		*17,632*			*17,632*	
Purchasing and preparing SMC drugs	59,495,789	122,060	35.91%	59,495,789	122,060	34.14%
Training	6,674,267	13,693	4.03%	8,093,772	16,605	4.64%
Equipment	17,251,396	35,393	10.41%	17,251,396	35,392	9.90%
Delivering to distribution point	374,863	626	0.23%	382,512	785	0.22%
Dispensing	74,073,986	134,752	44.71%	82,933,503	170,144	47.59%
IEC^a^	3,209,310	6,584	1.94%	3,209,310	6,584	1.84%
Total	165,690,044	316,111	100%	174,254,252	357,494	100%

^a^Information Education & Communication

The average financial unit costs per child per round was US $0.73. The economic costs by round of SMC and per child are shown in Table 5. The economic costs by round were similar for all the rounds except round 1, where the number of children reached was lower compared to the other rounds. On average, SMC cost $0.86 (424.986 FCFA) per child/per round. Adding the averages across the four rounds gives a cost, on average, of $3.43 per child for the annual economic cost per child receiving SMC. However, this does not take into account adherence. In terms of how the costs changed relative to adherence, the cost of a *fully adherent* child having received all four rounds was US $6.38. (i.e. total economic costs divided by the 53.8% of 104,255 children estimated to have received at least the first day treatment of all four SMC rounds in the trial [13]) There is evidence to suggest that receiving even three rounds of SMC provides substantial protection [31, 32] which would make the cost per child *highly adherent* US $4.69.

**Table 5:** Economic cost of SMC per rounds and accounting coverage in Kita (US $ 2016)

	1^st^ round		2nd round		3rd round	4th round		Total
Cost (US $)	90,526		88,345		89,466	89,157		357,494
Total Number of Individual Children reached	98,790		104,477		108,659	105,093		
Cost per child per round	0.92		0.85		0.82	0.85		3.43

Adherence description^a^		Coverage rate		Cost per child accounting for adherence rates				
Received at least the first day treatment of SMC in all 4 rounds		53.8%		US $ 6.38				
Received at least the first day treatment of SMC in at least 3 rounds		73.1%		US $ 4.69				
Received at least the first day treatment of SMC in at least 2 rounds		84.8%		US $4.05				

^a^Based on information from maternal interview only Taken from Diawara et al. [13]. Table 1 Coverage of SMC defined as proportion of children who received SMC drugs at day 1 and days 1–3 according to the source of the information (interview or SMC card) during the coverage survey and Prof Alassane Dicko personal communication

### Effectiveness

**Fig. 2:**
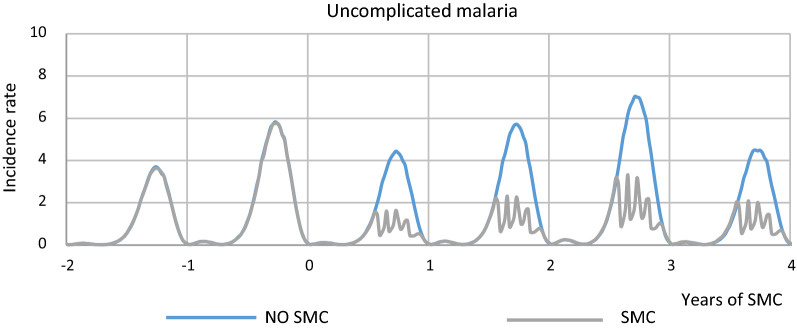
Uncomplicated malaria incidence in children under 5 with SMC and no SMC in Kita district

**Fig. 3:**
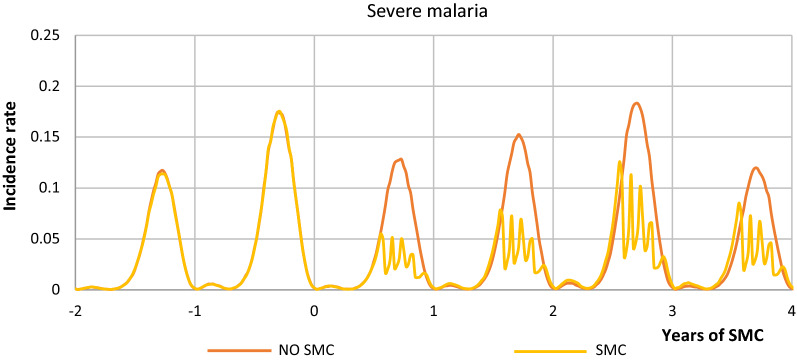
Severe malaria incidence in children under 5 with SMC and no SMC in Kita district

**Fig. 4:**
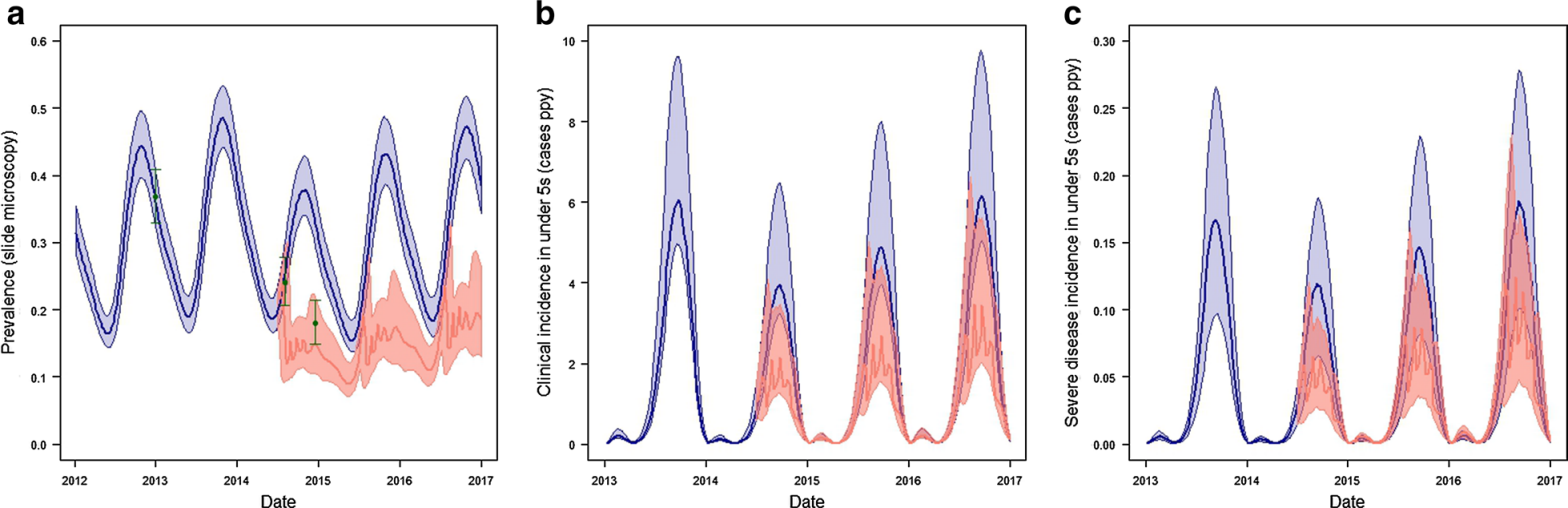
Simulations of the effectiveness of SMC in Kita shows **a** simulations of the impact of SMC upon prevalence in ages 3 -59 months, with estimates from prevalence surveys, including the 2014 pre-intervention to which the simulations were calibrated are shown as green dots with bars showing binomial 95% confidence intervals, **b** simulated incidence of uncomplicated clinical malaria in children under 5 and **c** incidence of severe malaria in children under 5. In all plot simulations using model parameters from the median of the joint posterior distribution and modal values from the triangle-distributed uncertainty distributions are shown in dark lines (blue for simulations in the absence of SMC, salmon in the presence of SMC), shaded areas show 95% uncertainty intervals based upon the 1000 simulated parameter draws. * Note these are 1 year averages based on a 3 year cycle that takes into account the waning protective efficacy of LLINs

Figures 2, 3 shows the range of model simulations for transmission in Kita prior to SMC and during SMC implementation, as well as counter-factual simulations for the trajectory of transmission and patterns of burden that would have occurred in the absence of SMC. Simulations were calibrated to the pre-intervention survey, LLIN scale-up and seasonal rainfall; show a good fit to prevalence measured soon after the end of the 2012 transmission season; and are within the range of the prevalence observed soon after the end of the intervention (Fig. 4). Counter-factual simulations capture the likely reductions in burden that will have occurred following the LLIN campaign in 2014, with transmission increasing towards the end of the 3-year LLIN distribution cycle. In the year of SMC implementation our simulations are consistent with SMC preventing 660 (435–922 95% uncertainty interval (UI)) uncomplicated cases and 15.4 (8.42–24.3 95% UI) cases of severe malaria per 1000 children reached among children below five years of age. In the all-age population, which also captures the impact of SMC on transmission, this represents 118 (78.2–163 95% UI) cases and 2.61 (1.43–4.14 UI) severe cases per 1000 person-years. Averaged over the 3-year LLIN distribution cycle this gives 804 (478–1213 95% UI) uncomplicated cases and 16.5 (8.89–27.1 95% UI) cases of severe malaria per 1000 children reached and 141 (84.3–209 95% UI) cases and 2.73 (1.46–4.43) severe cases per 1000 person-years. Table 6 provides the number of cases averted annually incorporating population size and these figures inform the ICER.

**Table 6:** Effectiveness outcomes: malaria episodes averted

Total over 1 year time horizon^a^ Kita pop	Children			All population		
	Cases	Uncomplicated	Severe	Cases	Uncomplicated	Severe
Mean	62,313	61,031	1282	72,676	71,265	1411
Lower 95% limit^a^ (based on 1000 simulations)	37,047	36,358	689	43,535	42,781	754
Higher 95% limit (based on 1000 simulations)	94,004	91,904	2099	108,144	105,853	2291

^a^Uncertainty bound

### ICER

The incremental cost effectiveness ratios (ICER) per malaria episode averted were estimated for children and for the entire population (Table 7). Based on predicted coverage, the financial cost per childhood episode averted was estimated at US $5.07 (uncertainty bounds 3.36–8.53) and the economic cost was US $5.74 (3.80–9.65), respectively. In the all-ages population in Kita, the economic cost per malaria episode averted was US $4.92 (range 3.31–8.21). Given that coverage was higher than predicted, it could be argued that the more accurate ICERs are a financial cost per childhood episode averted of US $3.77 (uncertainty bounds 2.50–6.34) and an economic cost of US $4.26 (range 2.83—7.17) per childhood episode averted. As shown in Table 7, the ICER is US $144 (135–153) per DALY averted and US $14,503 (13,604–15,402) per death averted based on actual children covered. See Appendix 1 for DALY calculations.

**Table 7:** Cost effectiveness analysis of SMC (US $ 2016)

	Mean	Lower 95% limit^a^	Higher 95% limit
Costs Per Episode Averted			
Financial cost per childhood episode averted (actual coverage)	3.77	2.50	6.34
Economic cost per childhood episode averted (actual coverage)	4.26	2.83	7.17
Financial cost per childhood episode averted (predicted coverage)	5.07	3.36	8.53
Economic cost per childhood episode averted (predicted coverage)	5.74	3.80	9.65
Financial cost per all ages episode averted (predicted coverage)	4.35	2.92	7.26
Economic cost per all ages episode averted (predicted coverage)	4.92	3.31	8.21
Economic Cost per DALY averted (actual coverage)	144	135	153
Economic Cost per Death averted (actual coverage)	14,503	13,604	15,402

^a^Uncertainty bounds – for cost per episode these were taken from the ranges of the model effectiveness estimates. For the DALYs and Deaths a ± 20% was used in the absence of any cost uncertainty, a gamma distribution was assumed for all parameters and 10,000 iterations were run using monte carlo simulations

## Discussion

The economic cost of SMC was US $0.86 per child per SMC round and the cost per child fully adherent was $6.38. The cost effectiveness ratios were estimated at an economic cost of US $4.26 per childhood episode averted and US $144 per DALY averted. Thus, SMC was cost effective as US $144 per DALY averted was well below two very conservative thresholds: (i) USD $779.94 per DALY averted, based on Mali’s 2016 GDP per capita [33] and (ii) an even more stringent World Bank threshold of $250 per DALY averted [29, 30].

In addition to the multi-country ACCESS SMC study (which included Mali) (12), other studies of routine SMC implementation comparable to Kita in terms of scale and their attempt to deliver through the routine health system have been conducted in Senegal [7], Upper West Region of Ghana [6], Koutiala in Mali [8], and elsewhere (see Table 1). At US $0.86 per child per round, Kita SMC unit costs are amongst the lowest reported to date. Only Senegal, where the economic cost per child per round was reported to be $0.55, shows a lower economic cost per round. A major difference in the Kita study and the Senegalese study is that costs incurred at national level were not included in the total Senegalese unit cost. Kita SMC delivery unit costs were lower than the two Malian SMC studies conducted to date. In Koutiala, an estimate based on the budget expenses found a unit cost per child per round at 1.12 euros or $1.30 (inflated to 2016 $US) [8], compared to an average economic cost of US $ 0.86 per round in Kita. The other Malian study, ACCESS-SMC, suggests a preliminary average estimates of $4.05 (USD 2015) for the delivery of four doses using both door-to-door and fixed point delivery strategies [10, 11] compared to US $3.43 delivery of four rounds in Kita. The average cost of four treatments per child, weighted across the seven ACCESS countries was US $3.63 (12).

This study shows drug costs and personnel costs (largely made up of incentives) were the main cost drivers. This is in line with most previous SMC cost studies, for example in the Senegal study incentives to CHWs and drugs costs were the highest costs, representing 44% and 27% of the total cost, respectively. The economic cost estimate of US $4.26 per malaria case averted (2.83–7.17), is lower than that found in Hohoe Ghana US $24.87 (22.63–27.36) per case averted and Upper West Region Ghana US $108.41 (101.01–123.01). However, the per death were higher than Upper West Region Ghana. At US $144 per DALY averted it is well below both conservative thresholds we enlisted to identify highly cost effective interventions. This is the first SMC cost-effectiveness analysis to use primary cost data to estimate the cost per DALY averted for SMC and also one of very few large-scale implementation studies to estimate the cost- effectiveness of SMC using a fixed point distribution.

The study has limitations. While the costs were taken directly from the intervention site, the effectiveness of the intervention was modelled on data drawn from surveys as data on malaria episodes and deaths averted were not available. However, the model was calibrated to local data and has been widely published. In terms of generalising beyond this study setting, SMC drugs were not in co- blister packs, so had to be packed manually and this resulted in additional costs. Using co-blister drugs, depending on their price, might lead to an increase the cost of the intervention.

## Conclusion

SMC was highly cost effective when delivered at large scale, fixed-points through the routine health system in Mali. The main costs were drugs and personnel, with financial incentives as the largest component of the personnel cost.

## Data Availability

The datasets analysed for this study are available from the corresponding author on reasonable request.

## Appendix 1: DALY and deaths averted calculations

DALYs for a disease or health condition are calculated as the sum of the Years of Life Lost (YLL) due to premature mortality in the population and the Years Lost due to Disability (YLD) for people living with the health condition or its consequences^,^^1^,^2^:

$$ {\text{DALY}} = {\text{YLL}} + {\text{YLD}}.$$

These calculations incorporate weights for life expectancy, age, future time and disability. See the table below for the weightings assigned to our analysis.^3^

$$ \begin{gathered}   {\text{Years}}\;{\text{of}}\;{\text{life}}\;{\text{lost}}\;({\text{YLD}}) \hfill \\   {\text{YLD (r, K, }}\beta {\text{)}} = D\left\{ {\frac{{KCe^{{ra}} }}{{(r + \beta )^{2} }}\left\{ {e^{{ - (r + \beta )(L + a)}} [ - (r + \beta )(L + a) - 1] - e^{{ - (r + \beta )a}} [ - (r + \beta )a - 1]} \right\} + \left( {\frac{{(1 - K)}}{r}} \right)(1 - e^{{ - rL}} )} \right\} \hfill \\  \end{gathered}  $$

where K: age weighting modulation factor, C: constant, r: discount rate expressed as decimal, a: age of onset disability, β: parameter from the age weighting function, L: duration of disability, D: disability weight.

Inputs

**Table:**

Formula	Malaria DALY Calculations and weightings		Assumption and source
	Uncomplicated	Severe	
	DALYs	DALYs	
C	0.1658	0.1658	
K	0	0	No age weighting applied
r	0.03	0.03	A discount rate of 3% applied
a	2.5	2.5	Mid point between sample cohort of 0–5
B	0	0	No age weighting applied
LD (7 or 30 days)	0.019178082	0.082191781	The assumed duration of an uncomplicated malaria episodes was 7 days, expressed in years (7/365) and a severe episode, 30 days, expressed in years (30/365)
D	0.006	0.133	IHME, GBD 2016–2017 (based on 2015 estimates at the most recent weightings available)
Life expectancy	72	72	According to the Global Health Observatory 72.0 years was the average life expectancy at birth of the global population in 2016 https://www.who.int/gho/mortality_burden_disease/life_tables/situation_trends/en/
CFR malaria	0.3%	0.3%	WHO Health Information 2015—Indicators https://www.who.int/healthinfo/indicators/2015/chi_2015_33_mortality_malaria.pdf
DALY per episode using the above parameters			
YLD	0.019	0.082	
YLL		30.31	

The total number of DALYs was calculated by multiplying the DALYs weights with the annual sum of clinical and severe episodes predicted in the epidemiological model
